# The Biochemistry and Epigenetics of Epilepsy: Focus on Adenosine and Glycine

**DOI:** 10.3389/fnmol.2016.00026

**Published:** 2016-04-13

**Authors:** Detlev Boison

**Affiliations:** Robert Stone Dow Neurobiology Laboratories, Legacy Research InstitutePortland, OR, USA

**Keywords:** epilepsy, epileptogenesis, adenosine, adenosine kinase, glycine, glycine transporter 1, epigenetics, DNA methylation

## Abstract

Epilepsy, one of the most prevalent neurological conditions, presents as a complex disorder of network homeostasis characterized by spontaneous non-provoked seizures and associated comorbidities. Currently used antiepileptic drugs have been designed to suppress neuronal hyperexcitability and thereby to suppress epileptic seizures. However, the current armamentarium of antiepileptic drugs is not effective in over 30% of patients, does not affect the comorbidities of epilepsy, and does not prevent the development and progression of epilepsy (epileptogenesis). Prevention of epilepsy and its progression remains the Holy Grail for epilepsy research and therapy development, requiring novel conceptual advances to find a solution to this urgent medical need. The methylation hypothesis of epileptogenesis suggests that changes in DNA methylation are implicated in the progression of the disease. In particular, global DNA hypermethylation appears to be associated with chronic epilepsy. Clinical as well as experimental evidence demonstrates that epilepsy and its progression can be prevented by biochemical manipulations and those that target previously unrecognized epigenetic functions contributing to epilepsy development and maintenance of the epileptic state. This mini-review will discuss, epigenetic mechanisms implicated in epileptogenesis and biochemical interactions between adenosine and glycine as a conceptual advance to understand the contribution of maladaptive changes in biochemistry as a major contributing factor to the development of epilepsy. New findings based on biochemical manipulation of the DNA methylome suggest that: (i) epigenetic mechanisms play a functional role in epileptogenesis; and (ii) therapeutic reconstruction of the epigenome is an effective antiepileptogenic therapy.

## Introduction

**Figure 1:**
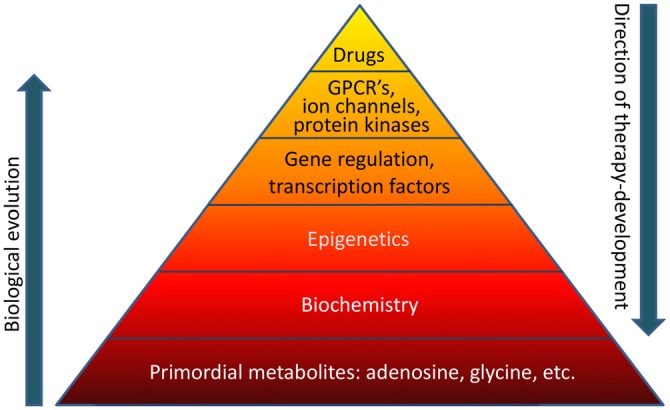
**The “pyramid of life”.** Evolutionary complexity started with key metabolites and biochemical mechanisms, which form the basis of all forms of life. In contrast, conventional drug development follows a top-down approach. GPCR’s, G protein coupled receptors.

Biological evolution is thought to have started with relatively simple, versatile, and multifunctional metabolites (Miller and Urey, 1959a,b). Adenosine was likely part of the “primordial soup” at the origin of life on Earth (Oro, 1961). Therefore it is not surprising that adenosine is an integral component of ATP, RNA (including poly-A tails), NAD, and other compounds essential for basic biochemistry and mitochondrial bioenergetics. Glycine in turn is the most primitive amino acid, which has additional biochemical functions in carbon metabolism. It is highly likely that the biochemical and epigenetic control of genes through global biochemical regulation preceded the “invention” of transcription factors, which later assumed the role to fine-tune a preexisting primordial biochemistry-based regulatory system. For example, an energy crisis would lower ATP needed for RNA synthesis and increase adenosine, thereby promoting increased DNA methylation as will be discussed in this mini-review in more detail. Both processes would reduce gene transcription globally and conserve energy. Therefore, primordial regulatory networks acting on a global homeostatic level likely preceded the “invention” of gene specific mechanisms that require sophisticated control through transcription factors, which in turn are regulated by G protein coupled receptors (GPCRs) and protein kinase pathways. In contrast, therapy development conventionally starts with the pharmacology of drugs. For example, benzodiazepines were almost discovered by chance in 1957 leading to the subsequent characterization of the “benzodiazepine receptor” in the CNS in 1977. It turned out that the benzodiazepine binding site was in fact an integral part of the GABA_A_ receptor complex (Möhler and Okada, 1977). Drug-driven therapy development led to a major focus on “druggable” GPCRs, ion channels, and protein kinases, which still form the main-stay of CNS therapeutics today. Methods to exploit gene regulation therapeutically are still in its infancy and the therapeutic potential of epigenetic, biochemical, and metabolomic approaches constitutes a new frontier in therapy development. If the basis of the pyramid depicted in Figure 1 is overlooked, it becomes obvious that a traditional pharmacological top-down treatment approach has limitations.

## The Biochemistry of Epilepsy

In the following, I will focus on temporal lobe epilepsy (TLE), the most common form of epilepsy in adult patients, and the most thoroughly studied form of epilepsy in animal models, as well as on two key metabolites, adenosine and glycine, whose homeostasis is known to be affected in the epileptic brain. Adenosine, primarily through activation of adenosine A_1_ receptors, is an endogenous anticonvulsant and seizure terminator of the brain (Dragunow, 1991; During and Spencer, 1992) that also controls a wide range of cognitive and psychiatric phenotypes (Boison et al., 2012). In human surgical specimens, as well as in rodent models of TLE, overexpression of adenosine kinase (ADK) and resulting adenosine deficiency associate with astrogliosis and constitute a pathological hallmark of TLE (Riban et al., 2002; Gouder et al., 2004; Fedele et al., 2005; Boison, 2008; Li et al., 2008, 2012; Aronica et al., 2011, 2013). Consequently, therapeutic adenosine augmentation effectively suppresses seizures in a wide range of rodent models of epilepsy (Huber et al., 2001; Zuchora et al., 2001; Gouder et al., 2003; Anschel et al., 2004; Vianna et al., 2005; Li et al., 2007b, 2008; Wilz et al., 2008; Boison, 2009, 2012a; Boison and Stewart, 2009; Van Dycke et al., 2010). A focus of this mini-review however is the underappreciated biochemical adenosine receptor (AR) independent effects of adenosine that are tightly linked to the control of DNA methylation and that are under the control of ADK, an enzyme which also has a specific isoform expressed in the nucleus of cells (Boison, 2013). The cytoplasmic isoform of the enzyme is thought to regulate the homeostatic pool of adenosine thereby determining the level of AR activation (Boison and Aronica, 2015), whereas the nuclear isoform of the enzyme strongly affects DNA methylation status (Williams-Karnesky et al., 2013). Interestingly, ADK undergoes biphasic expression changes during epileptogenesis in modeled TLE (Gouder et al., 2004; Li et al., 2008; Boison, 2013), which form the basis of the ADK hypothesis of epileptogenesis: Acute insults to the brain such as traumatic brain injury (Clark et al., 1997), seizures (During and Spencer, 1992; Gouder et al., 2004), or a stroke (Pignataro et al., 2008) lead to an acute surge in adenosine associated with transient downregulation of ADK within the first 2 to 3 h after the injury (Gouder et al., 2004; Pignataro et al., 2008). This acute phase is followed by a “latent period” of epileptogenesis, which occurs during the first few days or weeks after an insult in rodent models, or weeks and months in humans. During this latent period, inflammatory processes are activated that lead to microglial and astroglial activation (Nabbout et al., 2011; Vezzani et al., 2011; Devinsky et al., 2013). Astrogliosis is associated with increases in ADK expression and consequential development of adenosine deficiency. We have previously shown that seizures originate in areas of astrogliosis with overexpression of ADK (Li et al., 2008, 2012), that seizure onset during epileptogenesis temporally coincides with the emergence of astrogliosis and overexpressed ADK (Li et al., 2007a), that overexpression of ADK as such is sufficient to generate partial seizures (Li et al., 2007a, 2008), and that overexpression of ADK triggers hypermethylation of DNA (Williams-Karnesky et al., 2013). Since therapeutic adenosine augmentation restores normal DNA methylation levels and prevents epilepsy progression long-term (Williams-Karnesky et al., 2013) increased ADK and increased DNA methylation status might form a vicious cycle implicated in the progression and maintenance of epilepsy. Therefore, dysregulation of ADK appears to play a significant role in the processes that turn a normal brain into an epileptic brain.

**Figure 2:**
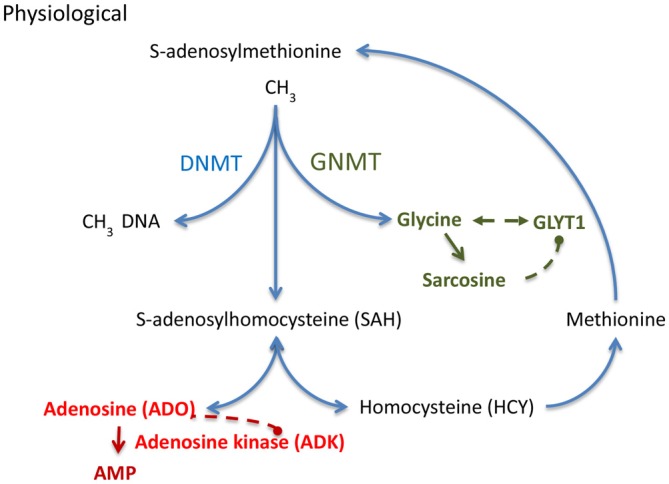
**S-adenosylmethionine (SAM) dependent transmethylation pathway, which is under the control of adenosine and glycine.** Adenosine and glycine are regulated by adenosine kinase (ADK) and glycine transporter 1 (GlyT1), respectively. DNMT, DNA methyltransferase; GNMT, glycine N-methyltransferase.

In the hippocampal formation, glycine can exert opposing effects that depend on the activation of presynaptic (Kubota et al., 2010; Winkelmann et al., 2014) vs. postsynaptic glycine receptors (GlyRs; Aroeira et al., 2011). It has recently been demonstrated (Chen et al., 2014) that low concentrations of glycine (10 μM) exert pro-convulsive effects, whereas higher glycine concentrations (100 μM) attenuate recurrent epileptiform discharges. The pro-convulsive actions of presynaptic GlyRs expressed on glutamatergic synapses (Winkelmann et al., 2014) is further supported by findings showing that the expression of edited GlyR encoding mRNAs are increased in the human epileptic hippocampus (Eichler et al., 2008) and that GlyR RNA editing regulates glycine affinity (Meier et al., 2005). These findings suggest that glycine homeostasis plays a crucial role in maintaining the balance between increased and decreased neuronal excitability. Hippocampal glycine is largely regulated by its reuptake transporter, glycine transporter 1 (GlyT1), found in both excitatory neurons and astrocytes (Tsai et al., 2004; Aragón and López-Corcuera, 2005; Cubelos et al., 2005; Eulenburg et al., 2005; Martina et al., 2005; Betz et al., 2006). Consequently, the genetic deletion of GlyT1 increased synaptic glycine availability (Gomeza et al., 2003). Engineered mice with a genetic deletion of GlyT1 in forebrain were characterized by a decrease in hippocampal glycine uptake, an increase in hippocampal NMDAR function, and a wide spectrum of pro-cognitive effects (Yee et al., 2006; Möhler et al., 2008, 2011). Therefore, GlyT1 is considered a promising target for the treatment of cognitive symptoms in schizophrenia and several compounds have been tested in phase II and III clinical trials (Black et al., 2009; Singer et al., 2009; Möhler et al., 2011). A recent study (Shen et al., 2015) provided the first comprehensive analysis of GlyT1 dysregulation in chronic TLE. GlyT1 expression during epileptogenesis was characterized as a biphasic response with initial downregulation of GlyT1 after epileptogenesis-precipitating seizures followed by sustained pathological overexpression of GlyT1 in chronic epilepsy as demonstrated in two mechanistically different models of TLE in mice and rats (Shen et al., 2015). It was further demonstrated that human TLE is likewise associated with increased levels of GlyT1. Conversely, the pharmacological suppression of GlyT1 or the genetic ablation of GlyT1 in the hippocampus provided robust reduction of both acute as well as chronic seizure activity in three different model systems (Shen et al., 2015). Therefore, glycinergic regulation of network excitability is altered in epilepsy and GlyT1 presents a rational therapeutic target for the treatment of epilepsy. Pathological overexpression of GlyT1 in progressive epilepsy also implies altered interactions of GlyT1 with the transmethylation pathway (Figure 2), a novel hypothesis further discussed below.

## The Epigenetics of Epilepsy

The role of epigenetics in epilepsy development is a new and emerging research area (Garriga-Canut et al., 2006; Qureshi and Mehler, 2010; Kobow and Blümcke, 2012; Lubin, 2012; Henshall and Kobow, 2015). The fact that epigenetic changes might play a significant role at least in TLE is important because in contrast to genetic mutations, epigenetic changes are potentially reversible. The knowledge of epigenetic mechanisms implicated in the development of epilepsy provides a conceptual and mechanistic framework for the future development of epigenetic therapies tailored to prevent epilepsy (antiepileptogenic) or its progression (disease modifying). Currently used antiepileptic therapies fail to address the underlying causes of epilepsy and do not halt epileptogenesis (Löscher and Brandt, 2010). Epileptogenesis is characterized by a progressive increase in frequency and severity of spontaneous recurrent seizures (SRS). Several mechanisms are implicated in the epileptogenic cascade including neuro-inflammatory responses, neuronal cell loss, mossy fiber sprouting, aberrant connectivity, and gliosis coupled with adenosine dysfunction (Dudek et al., 2002; Seifert et al., 2010; Vezzani et al., 2011; Aronica et al., 2012; Boison, 2012b). One potential unifying factor behind many of the pathological changes in epileptogenesis may be epigenetic modifications, which are likely further potentiated by epileptogenesis itself (Qureshi and Mehler, 2010; Kobow and Blümcke, 2011). Epigenetic modifications, which alter gene transcription without modifying the underlying DNA sequence, are plastic and can respond rapidly in response to environmental cues, an important endogenous mechanism for the control of gene expression. Changes in histone acetylation and methylation, as well as changes in DNA methylation have been shown to occur in mature cells in the central nervous system (CNS; Ma et al., 2009; Feng et al., 2010). Importantly, these changes occur regularly and rapidly. Even a single episode of neural synchronization exceeding 30 s in the hippocampus induces DNA methylation-dependent alterations in transcription of immediate-early genes and initiates a cascade of transcription factors contributing to long-term circuit alterations (Nelson et al., 2008). Therefore, epigenetic modifications offer new therapeutic alternatives to interfere with epileptogenesis.

## The Methylation Hypothesis of Epileptogenesis

Although several epigenetic mechanisms such as histone modifications that involve the addition or removal of methyl or acetyl groups might be implicated in epileptogenesis (Henshall and Kobow, 2015), recent evidence points to a critical role of DNA methylation changes for the development and progression of epilepsy, which will be discussed in the following. Altered DNA methylation in the brain has been implicated in psychiatric, neurodegenerative, and neurological conditions, including epilepsy (Kobow et al., 2009; Ma et al., 2009; Feng et al., 2010; Martin and Wong, 2013; Masliah et al., 2013; Coppieters et al., 2014; Tremolizzo et al., 2014). The methylation hypothesis of epileptogenesis suggests that seizures by themselves can induce epigenetic modifications and thereby aggravate the epileptogenic condition (Kobow and Blümcke, 2011). Specifically, increased activity of DNA methylating enzymes as well as hypermethylation of DNA has been associated with the development of human and experimental epilepsy (Kobow et al., 2009, 2013; Zhu et al., 2012; Williams-Karnesky et al., 2013; Miller-Delaney et al., 2015). Thus, interference with DNA methylation offers novel conceptual opportunities to prevent epilepsy.

## Brain Homeostasis and the Control of DNA Methylation

DNA methylation status depends on the equilibrium of biochemical enzyme reactions that add methyl groups to cytidine groups in the DNA (5-methylcytidine, 5mC) catalyzed by DNA methyltransferases (DNMTs) and those that convert methyl groups to hydroxymethyl groups catalyzed by Ten-eleven translocation (TET) enzymes in preparation for active DNA demethylation. Here I will focus on those mechanisms that add methyl groups to DNA; those mechanisms are linked to the S-adenosylmethionine (SAM) dependent transmethylation pathway (Figure 2), which is under the control of adenosine and glycine regulated by ADK (Boison et al., 2002; Boison, 2013) and GlyT1 (Gomeza et al., 2003; Yee et al., 2006), respectively. DNA methylation requires the donation of a methyl group from SAM, a process that is facilitated by DNMTs. The resulting product, S-adenosylhomocysteine (SAH) is then further converted into adenosine and homocysteine (HCY) by SAH hydrolase (SAHH). Critically, the equilibrium constant of the SAHH enzyme lies in the direction of SAH formation (Kredich and Martin, 1977); therefore, the reaction will only proceed when adenosine and HCY are constantly removed (Kredich and Martin, 1977; Boison et al., 2002). If metabolic clearance of adenosine through ADK is impaired, SAH levels rise (Boison et al., 2002). SAH in turn inhibits DNMTs through product inhibition (James et al., 2002). Based on adenosine’s role as obligatory end product of DNA methylation, we conclude that an increase in ADK and the resulting decrease in adenosine, as seen in chronic epilepsy (Li et al., 2008; Masino et al., 2011), would drive increased global DNA methylation in the brain. This process may be amplified, because adenosine is an inhibitor of ADK (Boison, 2013). Therapeutic adenosine augmentation may thus effectively reverse pathological DNA hypermethylation and thereby prevent epilepsy progression. The recent discovery of glycine-N-methyltransferase (GNMT) in the hippocampus (Carrasco et al., 2014) suggests that the availability of hippocampal glycine also controls the SAM-dependent transmethylation pathway by competing for methyl-groups. Increased GlyT1 as seen in chronic TLE (Shen et al., 2015) is expected to affect DNA methylation through interference with glycine homeostasis. Interestingly, the methylation of glycine leads to the formation of sarcosine, which is an endogenous inhibitor of GlyT1 (Javitt, 2012). Therapeutic glycine augmentation (e.g., via diet) may thus effectively divert methyl groups to the formation of sarcosine and thereby reduce: (i) pathological DNA hypermethylation; (ii) ameliorate the effects of pathologically overexpressed GlyT1; and (iii) prevent epilepsy progression.

## Towards Epigenetic Therapies for Epilepsy Prevention

The antiepileptogenic potential of transient focal adenosine-delivery was tested in a rat model of systemic kainic acid (KA)-induced progressive TLE (Williams-Karnesky et al., 2013). Young male rats (130–150 g) received an acute dose of KA (12 mg/kg i.p.) to trigger status epilepticus (SE). Only rats that exhibited at least 3 h of acute convulsive SE were used further. Rats were subjected to continuous long-term monitoring to quantify seizure activity. Once rats had reached a stable rate of 3–4 SRS per week at 9 weeks post KA, the animals were randomized and each rat received bilateral intraventricular adenosine-releasing silk-implants, silk-only implants, or a corresponding sham treatment. Adenosine releasing implants were designed to transiently deliver a stable dose of 250 ng adenosine per brain ventricle per day restricted to 10 days of drug delivery (Szybala et al., 2009). Twenty four hours after the surgery, continuous video monitoring was initiated, maintained for 4 weeks, and resumed after a 4 week hiatus for an additional 4 weeks. In the control groups seizures continued to increase both in number and severity. In contrast, in recipients of adenosine-releasing implants, seizures were almost completely suppressed after polymer implantation. Remarkably, reduced seizure activity was maintained far beyond expiration of adenosine-release from the polymer for at least 12 weeks following implantation. Even at 12 weeks after implantation, seizure incidence was reduced by more than 70%. Importantly, and in line with prolonged reduction of seizures, mossy fiber sprouting at 21 weeks following KA was significantly attenuated in adenosine-treated rats compared to controls. In line with those profound antiepileptogenic effects, the transient delivery of adenosine restored normal DNA methylation status long-term. These data demonstrate that the transient delivery of adenosine is sufficient to restore normal DNA methylation status and to prevent epilepsy progression long-term (Williams-Karnesky et al., 2013).

## Conclusions and Outlook

The realization that a transient local dose of adenosine can have long-lasting antiepileptogenic effects based on shifting the transmethylation equilibrium through mass action may offer new therapeutic opportunities for small molecule ADK inhibitors. ADK inhibitors had been in pre-clinical development in the early 2000’s for the indications of seizure management in chronic epilepsy, the control of chronic pain and robust anti-inflammatory effects in chronic conditions (McGaraughty et al., 2005). Although highly efficient in preclinical models, further drug development was halted due to unacceptable side effects related to chronic drug dosing (McGaraughty et al., 2005). The first generation of ADK inhibitors was designed to augment beneficial AR dependent effects of adenosine by raising extracellular levels of adenosine, which are at the same time responsible for wide-spread systemic adverse effects of those agents. Of note, patients with inborn global ADK deficiency develop hepatic encephalopathy and a wide range of neurological symptoms (Bjursell et al., 2011), however it is currently not known whether those symptoms are a primary cause of ADK deficiency in the brain, or secondary to hepatic encephalopathy. Early drug development efforts created a bias for the identification of agents with preferential action on the cytoplasmic isoform of ADK. ADK inhibitors with a higher selectivity for the nuclear isoform of ADK might capitalize on the epigenetic effects of adenosine while minimizing AR-mediated adverse effects. In addition, the short term use of ADK inhibitors, over days as opposed to chronic sustained drug dosing, might be acceptable if long-lasting epigenetically based therapeutic benefits can be achieved. Challenges for drug development remain. It needs to be determined whether new therapeutic agents can enter the brain and whether a higher level of selectivity for specific isoforms of ADK can be achieved. Due to the different distribution of nucleoside transporters within the brain there might be opportunities for the development of cell-type or isoform selective therapies. Glycine modifying therapies might constitute an alternative avenue to affect DNA methylation and potentially epileptogenesis. However, it needs to be determined how the adenosine and glycine systems interact on the epigenetic level. As discussed in this mini-review, understanding the biochemistry of epileptogenesis might light to the development of novel forms of therapeutic intervention.

## Author Contributions

DB conceptualized and wrote the manuscript.

## Conflict of Interest Statement

The author declares that the research was conducted in the absence of any commercial or financial relationships that could be construed as a potential conflict of interest.
